# Potential mechanisms underlying the acute lung dysfunction and bacterial extrapulmonary dissemination during *Burkholderia cenocepacia *respiratory infection

**DOI:** 10.1186/1465-9921-11-4

**Published:** 2010-01-18

**Authors:** Luiz G Cunha, Maria-Cristina Assis, Gloria-Beatriz Machado, Ana P Assef, Elizabeth A Marques, Robson S Leão, Alessandra M Saliba, Maria-Cristina Plotkowski

**Affiliations:** 1 Departamento de Microbiologia, Imunologia e Parasitologia, Faculdade de Ciências Médicas, Universidade do Estado do Rio de Janeiro, Brazil; 2 Laboratório de Pesquisa em Infecção Hospitalar, IOC/FIOCRUZ, Rio de Janeiro, Brazil

## Abstract

**Background:**

*Burkholderia cenocepacia*, an opportunistic pathogen that causes lung infections in cystic fibrosis (CF) patients, is associated with rapid and usually fatal lung deterioration due to necrotizing pneumonia and sepsis, a condition known as cepacia syndrome. The key bacterial determinants associated with this poor clinical outcome in CF patients are not clear. In this study, the cytotoxicity and procoagulant activity of *B. cenocepacia *from the ET-12 lineage, that has been linked to the cepacia syndrome, and four clinical isolates recovered from CF patients with mild clinical courses were analysed in both *in vitro *and *in vivo *assays.

**Methods:**

*B. cenocepacia-*infected BEAS-2B epithelial respiratory cells were used to investigate the bacterial cytotoxicity assessed by the flow cytometric detection of cell staining with propidium iodide. Bacteria-induced procoagulant activity in cell cultures was assessed by a colorimetric assay and by the flow cytometric detection of tissue factor (TF)-bearing microparticles in cell culture supernatants. Bronchoalveolar lavage fluids (BALF) from intratracheally infected mice were assessed for bacterial proinflammatory and procoagulant activities as well as for bacterial cytotoxicity, by the detection of released lactate dehydrogenase.

**Results:**

ET-12 was significantly more cytotoxic to cell cultures but clinical isolates Cl-2, Cl-3 and Cl-4 exhibited also a cytotoxic profile. ET-12 and CI-2 were similarly able to generate a TF-dependent procoagulant environment in cell culture supernatant and to enhance the release of TF-bearing microparticles from infected cells. In the *in vivo *assay, all bacterial isolates disseminated from the mice lungs, but Cl-2 and Cl-4 exhibited the highest rates of recovery from mice livers. Interestingly, Cl-2 and Cl-4, together with ET-12, exhibited the highest cytotoxicity. All bacteria were similarly capable of generating a procoagulant and inflammatory environment in animal lungs.

**Conclusion:**

*B. cenocepacia *were shown to exhibit cytotoxic and procoagulant activities potentially implicated in bacterial dissemination into the circulation and acute pulmonary decline detected in susceptible CF patients. Improved understanding of the mechanisms accounting for *B. cenocepacia*-induced clinical decline has the potential to indicate novel therapeutic strategies to be included in the care *B. cenocepacia*-infected patients.

## Background

Over the last decades, *Burkholderia cenocepacia *has emerged as an important respiratory pathogen in the cystic fibrosis (CF) community. Pulmonary colonization/infection by these bacteria may persist for months or even years but a minority of patients exhibits a rapid clinical deterioration associated with severe respiratory inflammation, epithelial necrosis and invasive disease, a condition known as cepacia syndrome [1]. Despite intense research efforts, the detailed pathogenic mechanisms underlying this poor outcome of CF patients are not clear. *B. cenocepacia *ability to induce a marked release of proinflammatory mediators [2-4] is likely to contribute to lung damage and respiratory failure but whether bacterial isolates recovered from patients with poor clinical prognosis exhibit differential virulence profile has been so far poorly investigated.

Increasing evidences suggest that inflammation and coagulation are linked to and amplify each other. In clinical settings associated with exacerbated inflammatory response, uncontrolled activation of the coagulation cascade leads ultimately to inadequate fibrin deposition in host microvasculature [5]. In lungs, fibrin deposition has also been demonstrated in the alveolar and interstitial compartments [6,7]. Alveolar clotting processes compromise the lung gas-exchange barrier. Moreover, thrombin and fibrin degradation products may further activate neutrophils and fibroblasts, contributing to lung injury. Because the lungs of CF patients is characterized by a florid inflammatory response, we wonder whether alveolar clotting processes may be involved in the pathogenesis of pulmonary decline observed in a proportion of *B. cenocepacia-*infected CF patients.

Coagulopathy associated with inflammatory response depends most notably on enhanced expression of tissue factor (TF), the major physiological initiator of the coagulation cascade [8]. Besides being expressed on different cell types, TF can be released from cell surfaces and circulate in extracellular fluids as a soluble fluid-phase protein [9] or associated with microparticles [10] shed from cell membranes upon cell activation and/or damage. Because microparticles exhibit also anionic phosphatidylserine at their surface, they provide a catalytic surface promoting the assembly of the enzyme complexes of the coagulation cascade, contributing to the thrombogenicity of extracelular fluids [10,11].

Different pathogens have been shown to up-regulate TF expression on human cells [12-14], thereby enhancing their procoagulant potential but, to our knowledge, the ability of *B. cenocepacia *to modulate TF expression has not yet been investigated.

To address the deficiency in the knowledge of *B. cenocepacia *pathogenicity, in the present study we compared bacteria of the ET-12 epidemic lineage, that has been linked to the cepacia syndrome [15], with four *B. cenocepacia *clinical isolates (CI) recovered from the airways of CF patients with mild clinical outcome in their expression of virulence features potentially implicated in invasive disease and lung function decline: cytotoxicity towards airway epithelial respiratory cells and ability to induce a procoagulant state in the lung environment.

## Materials and methods

### Bacterial strains and culture conditions

*B. cenocepacia *strain J2315, a member of the virulent lineage known as electrophoretic type 12 (ET-12), was provided by the Pasteur Institute microorganisms depository. Clinical isolates (Cl-1 to Cl-4) were recovered from the airway secretions of four different CF patients and belong to *B. cenocepacia *subgroup IIIA. Samples obtained from the patients were processed as described previously [16]. Bacteria were grown on Trypticase Soy Broth at 37°C for about 18 h, harvested by centrifugation and resuspended in M-199-HEPES medium (Gibco BRL, Gaithersburg, MD, USA) containing 10% fetal calf serum (FCS) to A_660 nm _= 0.1, corresponding to about 10^8 ^colony forming units (CFU)/mL.

### Airway epithelial cell culture

Transformed human bronchial epithelial cells from the BEAS-2B cell line were cultured in M-199-HEPES medium containing 10% FCS and glutamine (complete medium), and seeded in 24-well tissue culture plates (0.4 × 10^5 ^cells per well). After 48 h, cells were infected at a multiplicity of infection of about 100 bacteria per cell. Bacteria were centrifuged (1,000 g for 10 min) onto the cell monolayers prior to incubation at 37°C for 1 h. Cells were then incubated with complete culture medium containing gentamicin (1 mg/mL) and ceftazidime (1 mg/mL) for additional 19 h, to eliminate infecting microorganisms, as reported [2]. Control non-infected cells were treated similarly.

### Detection of bacterial cytotoxicity

Bacterial cytotoxicity was determined by the assessment of cell staining with propidium iodide, a cell-impermeable nucleic acid binding dye that only permeates leaky cell membranes [17]. Briefly, control and infected cells were detached from the microplate wells with 0.05%trypsin-0.02% EDTA solution, pooled with spontaneously detached cells present in culture supernatants, centrifuged, ressuspended in PBS containing 1% bovine serum albumin (PBS-BSA 1%), incubated with propidium iodide at a final concentration of 2 μg/mL for 10 min and analyzes with a FACscalibur flow cytometer (Becton Dickinson, Mountain View, CA, USA).

### Detection of cell-associated TF

Control and infected cells were detached from the microplate wells as described above, fixed with 4% paraformaldehyde and saccharose in PBS, permeabilized with 0.01% Triton X-100 in PBS for 5 min, rinsed, incubated with an anti-TF-FITC complex (American Diagnostica, Stanford, CT, USA) and analyzed by flow cytometry.

### Detection of TF and procoagunlant activity in cell culture supernatant

The Imubind TF ELISA and Actichrome TF activity kits (American Diagnostica) were used to quantify TF and detect a procoagulant activity, respectively, in cell culture supernatants, according to the manufacturer's instructions.

### Detection of TF-containing microparticles

Cell culture supernatants from control and infected cultures were centrifuged at 1,200 g for 3 min, to remove cell debris, and then centrifuged at 17,500 g for 30 min at 15°C, to pellet microparticles. Pellets were washed, treated simultaneously with the anti-TF-FITC and annexin V-Alexa Fluor 647 (Molecular Probes, Eugene, OR, USA) complexes for 30 min in ice and washed once with PBS. Microparticles were resuspended in PBS-BSA 1% and analyzed for 1 min by flow cytometry. The region corresponding to shed microparticles was gated in side scatter *versus *fluorescent intensity dot plot representations by using, as reference, a mix of fluorescent beads (Megamix; Biocytex, Marseille, France) of diameters to cover the microparticles (0.5 μm and 0.9 μm), as described [14].

### Analysis of chromosomal *B. cenocepacia *DNA restriction profiles

Isolates were typed by pulse field gel electrophoresis (PFGE) as described [18], following digestion of intact genomic DNA with *Spe*I (Invitrogen). DNA fragments were separated on 1% (w/v) agarose gels in 0.5% TBE (Tris-borate-EDTA) buffer using a CHEF DRIII apparatus (Bio-Rad, Hercules, CA, USA) with 6 V/cm, pulsed from 0.5 to 25 s, for 18 h and 30 to 60 s, for 3 h at 14°C. Gels were stained with ethidium bromide and photographed under ultraviolet light.

### *In vivo *assays

Female 8-12 wk old Swiss mice were injected intraperitoneally with cyclophosphamide (150 mg/kg) to induce granulocytopenia and favour acute *B. cenocepacia *infection. After 48 h, mice were anesthetized with a mixture of ketamine (65 mg/kg) and xylazine (13 mg/kg) administered intraperitoneally and 5 × 10^7 ^CFU of each bacterial isolate in 50 μL of sterile LPS-free saline were instilled into their tracheas. Control mice were instilled with sterile LPS-free saline. After 24 h, mice were anesthetized for blood collection by intracardiac puncture (for bacteriological culture and the assessment of leukocyte concentration), and killed by intraperitoneal injection of sodium pentobarbital. Mice airways were then washed with 1 mL of PBS, their livers were excised, macerated and serially diluted with sterile saline. Bacterial load in liver parenchyma was determined by plating serial dilution of liver macerates on blood agar plates. Mice bronchoalveolar lavage fluids (BALFs) were analysed for total leukocyte and protein concentration (BCA Protein Assay kit, Pierce Biotechnology, Rockford, IL, USA), as well as for lactate-dehydrogenase (LDH) (Sigma-Aldrich, St Louis, MO, USA) and procoagulant activity (American Diagnostica). Animal handling were in accord with the guidelines of the Animal Ethics Research Committee of the State University of Rio de Janeiro (protocol # CEA/210/2007).

### Statistical analysis

Statistical analysis was performed using a one-way analysis of variance (ANOVA) with the Bonferroni's test to determine significant differences between groups, unless otherwise stated. P values < 0.05 were deemed to be significant.

## Results

### *B. cenocepacia *isolates differed in their cytotoxicity

**Figure 1:**
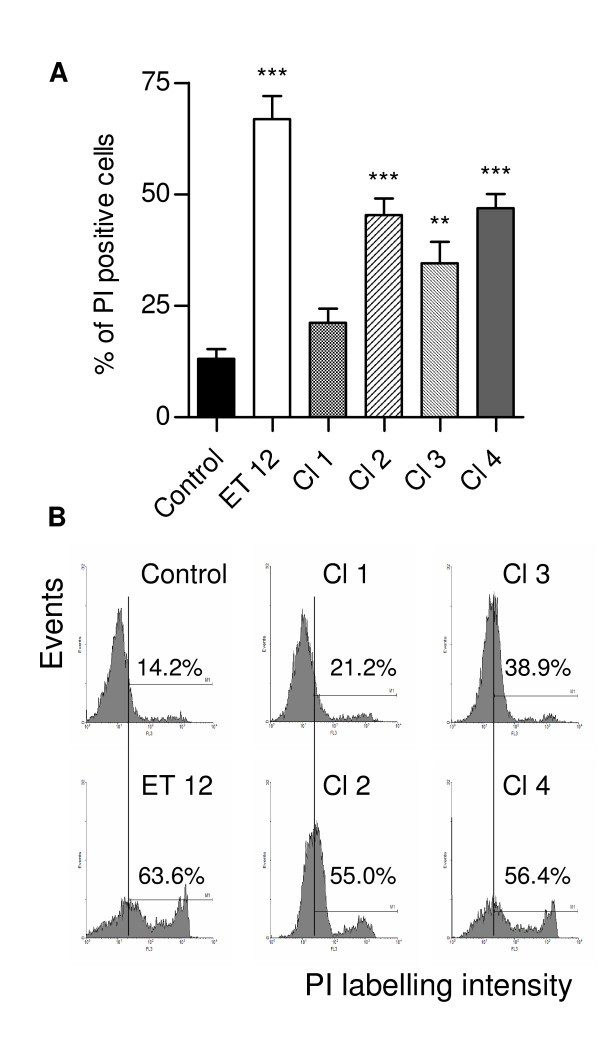
**Citotoxicity of *B. cenocepacia *assessed by FACS detection of cell staining with propidium iodide (PI)**. (A) Means (± SD) of the percentages of PI-stained cells detected in two assays carried out at least in triplicate. **, p < 0.01 and ***, p < 0.001 when data were compared with those from control non-infected cells; (B) Representative histograms showing the PI staining intensity of control and infected cells. y-axis corresponds to cell number whereas x-axis corresponds to log fluorescence intensity.

With the exception of Cl-1, all bacteria killed significantly high percentages of airway cells (Fig. 1). *B. cenocepacia *from the ET-12 lineage was shown to be significantly more cytotoxic than the other isolates (p < 0.001, 0.01, 0.001 and 0.05 when compared with Cl-1, Cl-2, Cl-3 and Cl-4, respectively).

### *B. cenocepacia *did not modify the expression of TF by infected cells but enhanced the release of TF into cell supernatants

**Figure 2:**
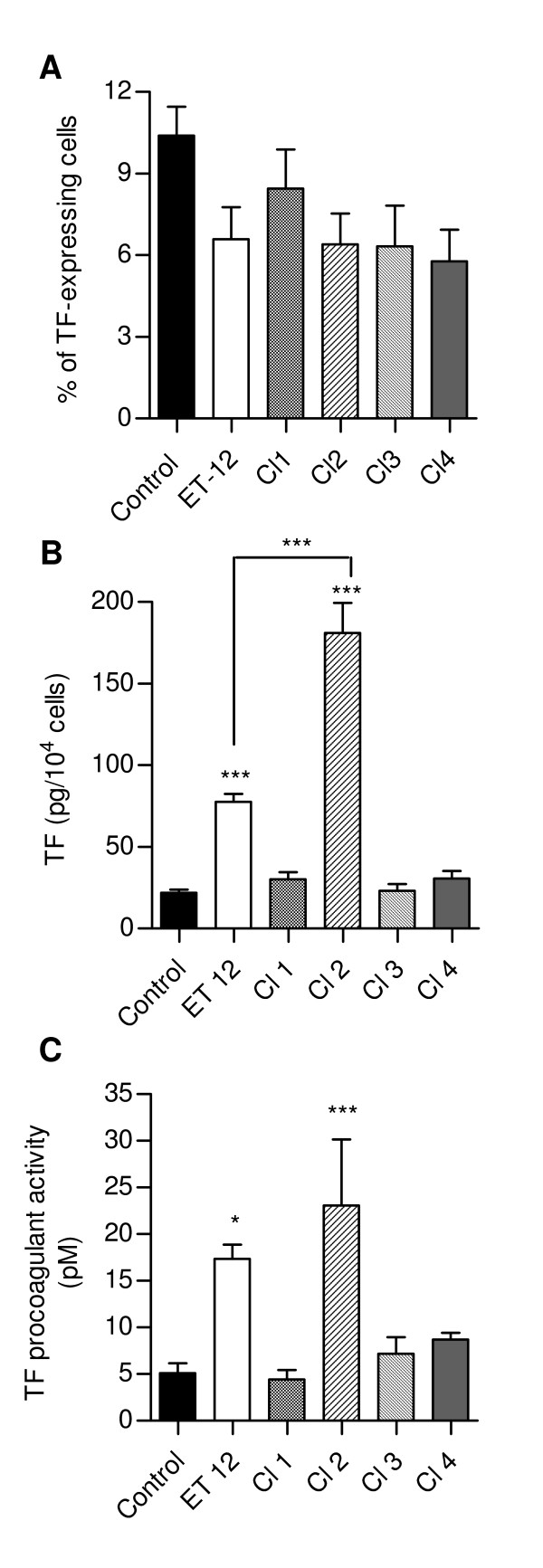
**Modulation of TF expression in infected cultures**. (A) Percentage of TF-expressing cells in control and infected cultures, determined by FACS analysis; (B) Concentration of TF in supernatants from control and infected airway epithelial cell cultures. (C) Procoagulant activity in cell culture supernatants. Data are means (± SD) of the results obtained in at least two different assays carried out in tripicate. *, p < 0.05 and **, p < 0.01 and p < 0.001 when data were compared with the results obtained with control non-infected cultures.

No significant difference between control and infected cultures in their percentage of TF-expressing cells could be detected (Fig. 2A), as well as their expression of TF mRNA (data not shown). In contrast, TF concentrations in supernatants from ET-12- and Cl-2-infected cultures were significantly higher than in supernatant from non-infected cultures and from cultures infected with the other clinical isolates, Cl-2 infection being the most important stimulus for TF release (Fig. 2B).

The biological relevance of released TF was next investigated. Fig. 2C shows that the supernatants from ET-12 and Cl-2-infected cells exhibited a significantly augmented procoagulant activity when compared with supernatants from control cultures and from cultures infected with the other clinical isolates (p < 0.01 for Cl-1 and p < 0.05 for Cl-3 and Cl-4).

### *B. cenocepacia *enhanced also the release of TF-bearing microparticles

**Figure 3:**
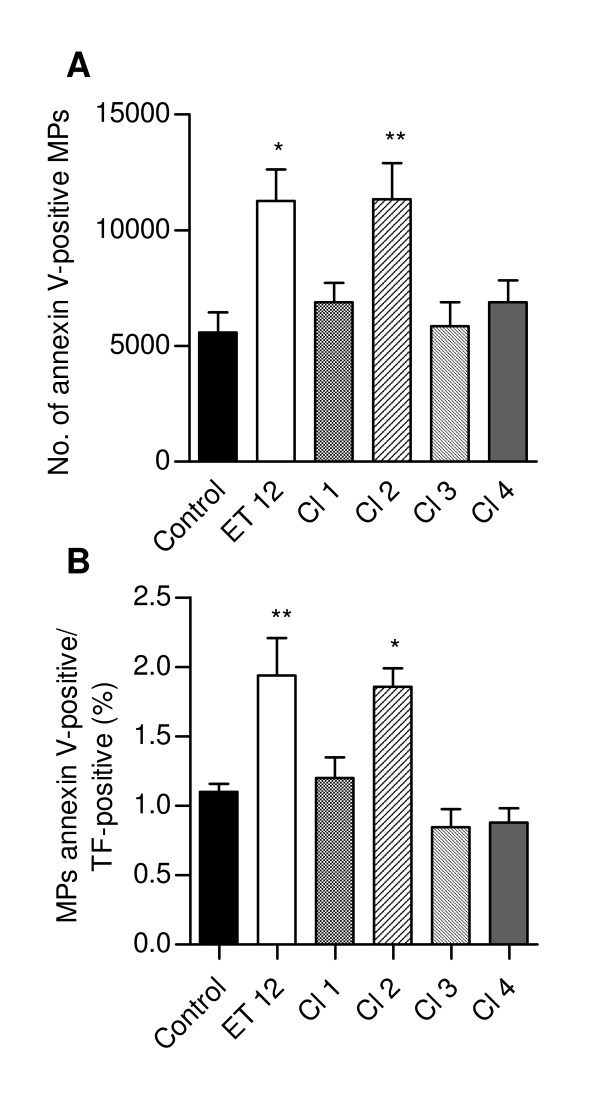
**Microparticle release from control and infected cells**. (A) Number of microparticles in control and infected cell culture supernatants submitted to FACS analysis for 1 min; (B) Percentage of TF positive/annexinV positive microparticles in supernatant from control and infected cultures. Data are means (± SD) of the results obtained in two assays carried out in triplicate. *, p < 0.05 and **, p < 0.01 when data from control and infected cells were compared with each other.

Fig. 3A shows that the number of microparticles binding annexin V, a protein known for its interaction with negatively charged phosphatidylserine residues, was significantly higher in supernatants from ET-12- and Cl-2-infected cells than in supernatants from control cultures. More importantly, a higher percentage of MPs shed after ET-12 and Cl-2 infection, besides reacting with annexin V, exhibited surface TF (Fig. 3B).

### Genetic relatedness of the *B. cenocepacia *isolates

**Figure 4:**
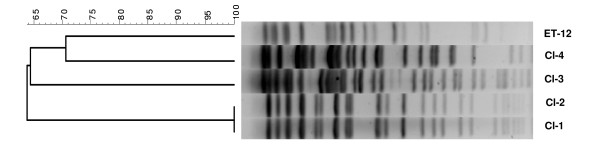
**PFGE profiles of genomic *B. cenocepacia *DNAs and dendogram resulting from computer analysis of PFGE profiles**.

Because ET-2 and Cl-2 exhibited a similar virulence profile, we wondered whether these two isolates were clonally related. However, PFGE analysis showed that all bacteria belonged to a different clonal group, with 70% maximum similarity, with the exception of Cl-1 and Cl-2 that exhibited exactly the same chromosomal DNA profile (Fig. 4).

### *In vivo *assays

**Figure 5:**
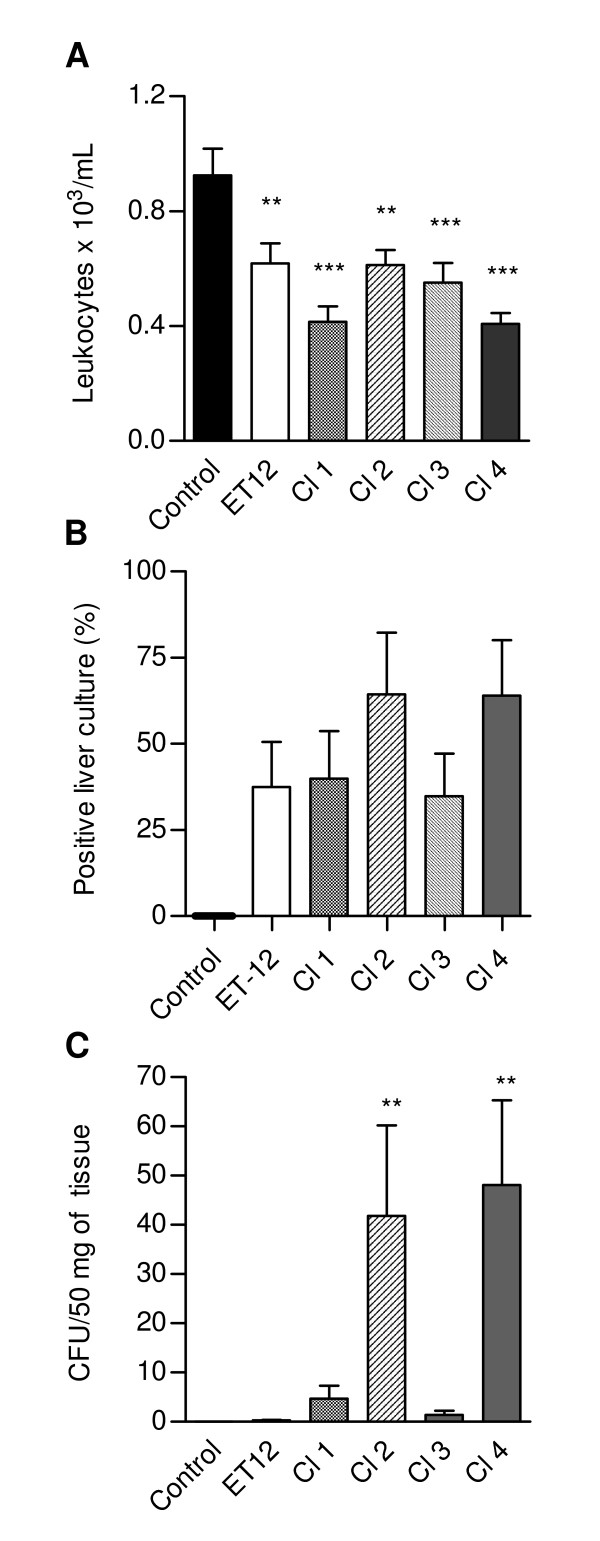
**(A) Blood leukocyte concentration in control and infected mice**. Data are means (± SD) of the results obtained in two assays in which at least 12 animals from each group were analysed. **, p < 0.01 and ***, p < 0.001 when data from control and infected mice were compared with each other. (B) Percentage of mice from each group with positive liver cultures; (C) Bacterial concentration in liver parenchyma. **, p < 0.01 when data from Cl-2- and Cl-4-infected mice were compared with data from the other groups by the Wilcoxon nonparametric test.

Total leukocyte concentrations in peripheral blood from mice infected with all clinical isolates were significantly lower than in blood from control mice, testifying the disease severity (Fig. 5A). All isolates were able to disseminate from the primary site of infection, as revealed by positive hemocultures for *B. cenocepacia *in all infected mice (data not shown). However, the percentages of Cl-2- and Cl-4-infected mice with positive liver cultures were higher than the percentages of mice infected with the other bacterial isolate, including ET-12, although the differences were not statistically significant (Fig. 5B). Bacterial concentration were also higher in liver parenchyma from Cl 2- and Cl 4-infected mice (Fig. 5C).

**Figure 6:**
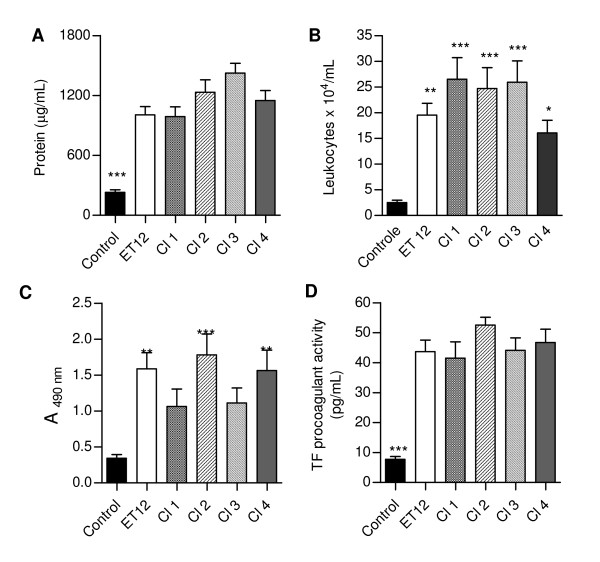
**(A) Total protein, (B) leukocyte, (C) LDH concentrations and (D) procoagulant activity in BALFs from control and infected mice**. Data are means (± SD) of the results obtained two assays in which at least 12 animals from each group were analysed *, p < 0.05, **, p < 0.01, ***, p < 0.001 when data from control and infected mice were compared with each other.

Infection with all *B. cenocepacia *isolates resulted in an inflammatory environment in mice lungs, revealed by significantly increased BALF concentrations of total protein and leukocyte (Fig. 6A and 6B, respectively). Most cells in BALFs from control mice were mononuclear (83.0% ± 15.9), whereas in fluids from infected animals most cells were polymorphonuclear (from 84.0% ± 10.6 to 96.1% ± 3.2). LDH concentrations in samples from infected mice were higher than in samples from control mice, testifying the bacterial cytotoxicity, although statistically significant differences were only detected when BALFs from control mice were compared with BALFs from ET-12-, Cl-2- and Cl-4-infected mice (Fig. 6C). BALFs from all infected mice exhibited a significant TF-dependent procoagulant activity (Fig. 6D).

## Discussion

Bacteria causing respiratory infections in CF patients typically remain confined to the endobronchial spaces. In contrast, a proportion of *B*. *cenocepacia*-infected patients exhibits an invasive disease, characterized by bacterial extrapulmonary dissemination and systemic inflammatory response [15]. The mechanisms that permit bacteria to disseminate are not yet known but are likely to involve penetration of airway barriers. *In vitro *studies provided evidences that *B*. *cenocepacia *from the ET-12 lineage can increase the permeability of and traverses polarized respiratory epithelium [19] by the dephosphorylation and dissociation of occludin from the tight-junction complex [20]. However, increase in epithelium permeability can also be secondary to epithelial cell death resulting in breachs of the epithelium barrier properties. Interestingly, evidences of damage to airway epithelial cells in culture were detected in areas subjacent to *B. cenocepacia *biofilms [19]. Cell damage was also detected in airway epithelial cell cultures infected with bacterial isolates carrying the cable pilin gene (21), a distinctive feature of *B. cenocepacia *from the ET-12 lineage [1]. More recently, purified cable pili were found to directly induce cytotoxicity in airway epithelial cells *in vitro *[22].

In the *in vitro assays *of this present study, besides ET-12, most clinical *B. cenocepacia *isolates were shown to kill airway epithelial cells but the specific virulence determinant and the corresponding genetic element required for *B*. *cenocepacia *cytotoxicity were not investigated. Interestingly, in the *in vivo *assay, clinical isolates accounting for the highest LDH concentration in mice BALF (Cl-2 and Cl-4) were recovered in higher frequency and concentrations in liver parenchyma. On the basis of these results, it is tempting to suggest a relationship between bacterial cytotoxicity and dissemination into the circulation. However, since such relationship was not detected in ET-12-infected mice, further studies are required to examine this hypothesis.

A huge inflammatory reaction is a hallmark of CF patient lung parenchyma [15]. Because inflammation almost invariably leads to the activation of the coagulation cascade [5], and intra-alveolar fibrin deposition plays a pathogenic role in lung dysfunction detected in many acute inflammatory lung diseases [23], we wondered whether *B. cenocepacia *would induce a procoagulant state in patient airspaces.

Increase of lung procoagulant state depends on enhanced expression of TF by airway cells followed by local TF-induced activation of the coagulation, in addition to being influenced by insufficiency of natural inhibitors of coagulation and of the fibrinolytic system [24]. Prominent among the proinflammatory stimuli known to modulate TF expression in monocyte and endothelial cells is bacterial LPS [25]. LPS was also shown to upregulate TF expression in lung tissues and fibrin deposition in the alveolar spaces, bronchioles and vessels of experimental animals [26]. Because *B. cenocepacia *LPS is a potent inducer of the inflammatory response [27,28], we were surprised to find no increase in TF expression in infected airway epithelial cells. A similar result was recently described in airway cell cultures infected with an ExoU-deficient *P. aeruginosa *strain [14]. Since most *in vitro *studies showing the regulatory effect of LPS on TF expression have been carried out with monocyte/macrophages or endothelial cells, we wonder whether the apparent contradiction between our results and the others may have stemmed from differential response of these several cell types. Alternatively, it is conceivable that the concentration of LPS released from infecting bacteria during the experimental assays may be much lower than the concentration of purified LPS used in those *in vitro *studies.

In contrast with the absence of modulation of TF expression at airway cells surface, significantly increased TF concentration and procoagulant activity were detected in supernatants from ET-12- and Cl-2-infected cells. These two bacterial isolates elicited also a significant release of TF-bearing microparticles from airway cells. Although the procoagulant activity detected in culture supernatants may have resulted from released soluble fluid-phase TF, it most likely resulted from the release of TF-bearing microparticle. This is so because TF requires association with anionic lipids to become procoagulant [29]. Whereas anionic lipids are not associated with soluble fluid-phase TF, they are constitutively expressed in microparticles. Studies showing that circulating TF-bearing microparticles are often associated with thrombotic propensity [10,11] corroborate our hypothesis.

Differences between the results from *B. cenocepacia-*induced procoagulant activity in cell culture supernatants and in mice BALFs are likely reflect the complexity of the *in vivo *experimental model in which different cell types are likely to contribute to the generation of the procoagulant activity. Because TF expression in cells from the monocytic lineage **IS **enhanced substantially upon cell activation, we wonder whether *B. cenocepacia-*stimulated alveolar macrophage may have contributed the generation of a potent TF-dependent procoagulant activity in mice BALFs, surmounting a milder response of airway epithelial cells.

In this report, in both *in vitro *and *in vivo *assays, Cl-2 was phenotypically similar to ET-12 *B. cenocepacia *but these two bacteria exhibited a very different PFGE profile. On the other hand, *B. cenocepacia *Cl-1 and Cl-2, that were indistinguishable by PFGE analysis, differed markedly in their virulence properties against airway cells.

*B. cenocepacia *possess very large genomes and separate their DNA into three or more chromosomal replicons which may add greater flexibility in the acquisition, loss and expression of genes [30,31]. Indeed, genome-sequencing projects have shown that 10% of more of the *Burkholderia *genes have been acquired through gene horizontal transfer and reside as elements of foreign DNA such as genomic islands, prophages or plasmids. Therefore, it is conceivable that genes encoding virulence factors accounting for the cytotoxicity and procoagulant activity of *B. cenocepacia *ET-12 and Cl-2 may reside as elements of foreign DNA that are not possessed by all *B. cenocepacia *isolates and were not detected by PFGE analysis. Similarly, foreign DNA elements possessed by Cl-2, but not by Cl-1, would explain why these two bacterial isolates, that exhibit the same chromosomal DNA profile, have different virulence phenotypes. Studies to examine this hypothesis are currently in progress.

## Conclusion

In this report, *B. cenocepacia *from the ET-12 lineage and clinical isolates were shown to exhibit virulence features potentially implicated in bacterial dissemination into the circulation and acute pulmonary decline detected in susceptible CF patients: cytotoxicity to airway epithelial cells, capability of enhancing the release of TF-bearing microparticles from infected cells and generating a TF-dependent procoagulant environment. *In vivo *assays corroborated the *B. cenocepacia *cytotoxicity as well as the ability to generate a procoagulant and inflammatory environment in mice airways.

Although differences between experimental models and humans preclude direct extrapolation of results from experimental studies to patients, on the basis of our *in vitro *and *in vivo *evidences we speculate that at least some *B. cenocepacia *isolates may be able to induce a prothrombotic state in CF patient airways, ultimately resulting in deposition of fibrin in airspaces. This hypothesis is supported by our recent demonstration of thrombus formation in lung parenchyma of *Pseudomonas aeruginosa-*infected mice with increased local procoagulant activity [14,32]. Besides compromising the lung gas-exchange barrier, airway clotting processes are harmful because surfactant components may be incorporated into polymerizing fibrin with subsequent loss of surface activity and alveolar instability, further contributing to lung function deterioration. Improved understanding of the mechanisms accounting for *B. cenocepacia*-induced procoagulant activity has the potential to indicate novel therapeutic strategies to be included in the care *B. cenocepacia*-infected patients.

## Competing interests

The authors declare that they have no competing interests.

## Authors' contributions

LGCJ performed most of the assays. MCA contributed to the design of the study and participated of all flow cytometry assays. GBM participated of all *in vivo *assays. APDCA, RSL and AMS participated of the molecular biology studies. EAM contributed to the design of the study. MCP conceived and coordinated the study, participated in statistical analysis and wrote the manuscript. All authors read and approved the final manuscript.
